# Supplementary material for: A unified drug–target interaction prediction framework based on knowledge graph and recommendation system
Source: Nat Commun. 2021 Nov 22;12:6775. doi: 10.1038/s41467-021-27137-3 (PMC8635420; doi:10.1038/s41467-021-27137-3)
Supplement: Supplementary file 1 — Supplementary Information [file 41467_2021_27137_MOESM1_ESM.pdf]

Supplementary Materials

1. Benchmark datasets

Supplementary Table 1. The number of the nodes and edges in the knowledge graph of the Luo's dataset.

Type of nodes	Count	Type of types	Count
Drug	708	Drug - Protein	1923
Protein	1512	Drug - Drug	10036
Disease	5603	Drug - Disease	199214
Side-effect	4192	Drug - Side-effect	80164
		Protein - Protein	7363
		Protein - Disease	1596745
Total	12015	Total	1895445

Supplementary Table 2. The number of the nodes and edges in the Hetionet.

Type of nodes	Count	Type of types	Count
Compound	1552	Anatomy–downregulates–Gene	102240
Gene / Proteins	20945	Anatomy–expresses–Gene	526407
Pathway	1822	Anatomy–upregulates–Gene	97848
Pharmacologic class	345	Compound–binds–Gene	11571
Biological Process	11,381	Compound–causes–Side Effect	138944
Cellular Component	1391	Compound–downregulates–Gene	21,102
Molecular Function	2884	Compound–palliates–Disease	390
Side effect	5734	Compound–resembles–Compound	6486
Symptom	438	Compound–treats–Disease	755
Disease	137	Compound–upregulates–Gene	18756
Anatomy	402	Disease–associates–Gene	12623
		Disease–downregulates–Gene	7623
		Disease–localizes–Anatomy	3602
		Disease–presents–Symptom	3357
		Disease–resembles–Disease	543
		Disease–upregulates–Gene	7731
		…	…
Total	47031	Total	2250197

We list the main type of nodes and edges in KG and more detailed information could be found in [1].

Supplementary Table 3. The number of the nodes and edges in the knowledge graph of the Yamanishi_08's dataset.

Type of nodes	Count	Type of types	Count
Drug	791	Drug - Protein	5127
Protein	989	Protein - Protein	4623
Pathway	1305	Drug - Pathway	12751
BRITE	56	Protein - Pathway	18766
Biological Process	3914	Drug - BRITE	3354
Cellular Component	452	Protein - BRITE	2572
Molecular Function	1249	Protein - Biological Process	12403
Motif	1221	Protein - Cellular Component	5199
Drug Group	687	Protein - Molecular Function	4875
Domain	534	Protein - Motif	4374
Binding site	19	Drug - Drug Group	2714
Active site	43	Protein - Domain	2204
Type	3	Protein - Binding site	153
Family	708	Protein - Active site	281
		Drug/Protein/Pathway - Type	1966
		Protein - Family	1584
…	…	…	…
Total	25487	Total	95579

We list the main type of nodes and edges in KG and more detailed information could be found in the Supplementary of Mohamed *et al.* 's work [2].

Supplementary Table 4. The number of the nodes and edges in the BioKG.

Type of nodes	Count	Type of types	Count
Compound	8805	Drug-Target Interaction	28033
Proteins	59145	Drug-Drug Association	1334085
Disease	5812	Protein-Protein Association	113817
Pathway	14688	Protein-Pathway Association	250118
		Protein-Disease Association	109276
		Drug-Disease Association	66867
…	…	…	…
Total	105524	Total	2043846

We list the main type of nodes and edges in KG and more detailed information could be found in [3]. It should be note that the number of DTI pairs in this study is 26051 since the structure characterization of some drugs and proteins is unavailable.

2. Experimental setup

1.1 Baseline methods

DeepDTI and MPNN_CNN: they are end-to-end methods based on deep learning. These models consist of two major steps: (1) generating feature vectors and then (2) applying deep learning to DTI prediction. The difference between these two methods is that DeepDTI utilizes sequence-based deep learning (CNN) and MPNN_CNN utilizes graph-based deep learning (MPNN) for drug coding. And the protein coding both utilizes CNN. Since the volume of the benchmark datasets is limited, we use the loss of the training set as the decision evidence for early stopping.

RF: The majority of machine learning methods for DTI prediction belong to the feature-based methods (also called proteochemometrics when both utilizing drug features and protein features), such as SVM, tree-based methods and other kernel-based methods. Among these methods, RF achieves satisfied predictive performance without consume much time.

DTINet: A computational pipeline to predict DTIs from a constructed heterogeneous network. DTINet first performs random walk with restart (RWR) on each network to obtain a distribution of each drug or protein node. Then, to cope with the noise derived from the network, DTINet applies the DCA method to approximate the obtained diffusion distribution by constructing a model parameterized based on a low-dimensional vector representation. This step is called compact feature learning and the resulting low-dimensional vector is called the feature vector. Lastly, DTINet computes the best projection from drug space onto protein space via a matrix completion method (also called inductive matrix completion). In this work, we used the feature vectors that DTINet supplies and then trains the inductive matrix completion model using these feature vectors on our split datasets.

DTiGEMS+: A method integrates different techniques form ML, graph embedding, graph mining and similarity-based methods. That is, (1) graph embedding was used in node2vec feature representation to benefit from the network topology and structural features, (2) graph mining was used to extract path score features, (3) similarity-based techniques were used to select and integrate multiple similarities from different information sources, and finally, (4) ML for classification. There are several classifiers like RF and MLP could be chosen for final prediction. In this work, we used the MLP

classifier as final training model and used the features supplied by DTiGEMS+.

TriModel: TriModel is a knowledge graph embedding model based on tensor factorization that extends the DistMult and ComplEx [2]. It represents each entity and relation using three embedding vectors such that the embedding of entity i is $\Theta_E(i) = \{e_i^1, e_i^2, e_i^3\}$ where all embedding vectors have the same size K (a user-defined embeddings size). Similarly, the embedding of relation j is $\Theta_E(j) = \{w_i^1, w_i^2, w_i^3\}$. e^m and w^m denote the m part of the embeddings of the entity or the relation, and $m \in \{1,2,3\}$ represents the three embeddings parts.

1.2 Feature preparation

Morgan fingerprints are calculated as 1024 bits. The class of CTD Protein descriptors has 147 descriptors describing different properties of amino acids. These descriptors are scaled to a range from 0 to 1 and then input to the models. Besides, the similarity matrix can also be employed as characterization of drugs and proteins. In RF, NFM and KGE_RFM, we used the best performed combination of the different kind features.

1.3 Training procedure of KGE_NFM

The training procedure mainly consists of two parts: KGE model training and NFM model training. Knowledge graph embeddings are learned by training a neural architecture over a graph. Although such architectures vary, the training phase always consists in assigning scores to triples by the model-specific scoring function and minimizing a loss function. Here, the dataset consisting of KG and training set is applied for KGE model training, in which the training set is applied as the observation dataset for early stopping. In the procedure of NFM model training, we also used the training set as the observation dataset for early stopping since the volume of the whole dataset is limited. When we split a validation set from the training set for early stopping, the training set is hardly optimized and the performance is bad. Thus, we tried to overcome this issue by observing on the training set with a strict regularizer.

1.4 Parameter settings

We use the grid search to optimize the model's hyperparameters.

Supplementary Table 5. Hyperparameters settings for each method.

Methods	Hyperparameters	Search space	Best parameters
MPNN_CNN	Hidden dimensions of classifier	[[256,256,128], [512,512,256], [1024,1024,512]]	[1024,1024,512]
	Learning rate	[0.1, 0.01, 0.001]	0.001
	Dimension of hidden layer of drug feature	[32, 64, 128]	128
	Hidden dimensions of MPNN	[32, 64, 128]	128
	Depth of MPNN	[1, 2, 3]	3
	The size of CNN filter when encoding protein	[[8,16,16], [32,64,64]]	[32,64,64]
	The size of CNN kernel when encoding protein	[[1, 2, 2], [4, 8, 8]]	[4, 8, 8]
	Batch size	Fixed due to the memory limitation	5000
DeepDTI	Hidden dimensions of classifier	[[256,256,128], [512,512,256], [1024,1024,512]]	[1024,1024,512]
	Learning rate	[0.1, 0.01, 0.001]	0.001
	The size of CNN filter when encoding drugs	[[8,16,16], [32,64,64]]	[32,64,64]
	The size of CNN kernel when encoding drugs	[[1, 2, 2], [4, 8, 8]]	[4, 8, 8]
	The size of CNN filter when encoding proteins	[[8,16,16], [32,64,64]]	[32,64,64]
	The size of CNN kernel when encoding proteins	[[1, 2, 2], [4, 8, 8]]	[4, 8, 8]
	Batch size	Fixed due to the memory limitation	5000
RF	The function to measure the quality of a split.	[Gini, Entropy]	Entropy
	The number of trees in the forest.	[100, 200, 500]	200
	The maximum depth of the tree.	[5, 10, None]	None
	Class weight	[None, Balanced]	Balanced
DTINet	Rank	[10, 30, 50]	50
	Regularization parameter lambda	[0.01, 0.1, 10]	0.1
	Number of iterations	[10, 30, 50]	Depends on the convergence situation, normally 10
DTiGEMS+	Hidden dimensions of classifier	[[64, 64, 64], [128128,128]]	[128, 128,128]
	Embedding space dimensionality	[300, 400, 500]	400

KGE (DistMult & TriModel)	Learning rate	[0.1, 0.01, 0.001]	0.001
	loss	[multiclass nll, absolute margin likelihood, pairwise]	pairwise
	Weight of regularization loss	[1e-3, 1e-4, 1e-5]	1e-5
	Regularizer norm	[1, 2, 3]	3
NFM	the layer number and units in each layer of deep net	[32, 64, 128]	[128]
	L2 regularizer strength applied to DNN	[1e-3, 1e-4, 1e-5]	1e-5 for warm start and 1e-3 for cold start
	L2 regularizer strength applied to linear part	[1e-3, 1e-4, 1e-5]	1e-5 for warm start and 1e-3 for cold start
	Learning rate	[0.1, 0.01, 0.001]	0.001
	Batch size	[5000, 10000]	10000

3. Evaluation performance

Supplementary Table 6. Evaluation performance on the Luo's dataset in the three challenging scenarios.

Metrics	Scenario	Ratio	End-to-end methods		Feature-based methods		Heterogeneous data driven methods	
			MPNN_CNN	DeepDTI	RF	NFM	DTINet	KGE_NFM
AUROC	Warm start	balanced	0.830	0.859	0.925	0.835	**0.940**	0.903
		unbalanced	0.929	0.952	0.953	0.923	0.944	**0.962**
	Cold start for drugs	unbalanced	0.806	0.662	0.791	0.741	0.853	**0.881**
	Cold start for proteins	unbalanced	0.431	0.487	0.484	0.755	0.778	**0.813**
AUPR	Warm start	balanced	0.805	0.840	0.932	0.825	**0.941**	0.898
		unbalanced	0.705	0.793	0.830	0.743	0.817	**0.855**
	Cold start for drugs	unbalanced	0.462	0.225	**0.607**	0.368	0.592	0.555
	Cold start for proteins	unbalanced	0.078	0.092	0.095	0.421	0.388	**0.444**

Supplementary Table 7. Evaluation performance on the Hetionet in the three sample scenarios.

Metrics	Scenario	End-to-end methods		Feature-based methods		Heterogeneous data driven methods	
		MPNN_CNN	DeepDTI	RF	NFM	TriModel	KGE_NFM
AUROC	Warm start	0.960	0.963	0.958	0.911	0.963	**0.972**
	Cold start for drugs	0.929	0.891	**0.934**	0.872	0.908	0.900
	Cold start for proteins	0.480	0.521	0.516	0.582	0.820	**0.878**
AUPRC	Warm start	0.721	0.756	0.739	0.610	0.746	**0.780**
	Cold start for drugs	0.641	0.564	**0.689**	0.563	0.554	0.577
	Cold start for proteins	0.082	0.096	0.086	0.139	0.250	**0.408**

Supplementary Table 8. Evaluation performance on the Yamanishi_08's dataset in the three sample scenarios.

Metrics	Scenario	Ratio	End-to-end methods		Feature-based methods		Heterogeneous data driven methods		
			MPNN_CNN	DeepDTI	RF	NFM	DTiGEMS+	TriModel	KGE_NFM
AUROC	Warm start	balanced	0.834	0.865	0.903	0.921	0.964	0.951	**0.968**
		unbalanced	0.974	0.982	0.949	0.939	0.976	**0.985**	0.983
	Cold start for drugs	unbalanced	0.629	0.628	0.832	0.720	0.745	0.817	**0.853**
	Cold start for proteins	unbalanced	0.502	0.497	0.467	0.780	0.674	0.829	**0.921**
AUPRC	Warm start	balanced	0.788	0.820	0.901	0.922	0.957	0.946	**0.961**
		unbalanced	0.874	**0.917**	0.776	0.773	0.874	0.886	0.902
	Cold start for drugs	unbalanced	0.194	0.191	**0.561**	0.330	0.518	0.503	0.521
	Cold start for proteins	unbalanced	0.098	0.099	0.117	0.454	0.443	0.483	**0.679**

Supplementary Table 9. Evaluation performance on the BioKG in the three sample scenarios.

Metrics	Scenario	End-to-end methods		Feature-based methods		Heterogeneous data driven methods	
		MPNN_CNN	DeepDTI	RF	NFM	TriModel	KGE_NFM
AUROC	Warm start	0.987	**0.988**	0.971	0.938	0.984	0.987
	Cold start for drugs	0.970	0.966	0.971	0.936	0.964	**0.978**
	Cold start for proteins	0.768	0.806	0.666	0.847	0.852	**0.899**
AUPRC	Warm start	0.889	**0.907**	0.891	0.769	0.876	0.898
	Cold start for drugs	0.871	0.844	**0.891**	0.764	0.839	0.881
	Cold start for proteins	0.245	0.341	0.132	0.303	0.392	**0.549**

Supplementary Table 10. Impact of each component in the KGE_NFM framework on the predictive performance in the scenario of the warm start in the unbalanced situation.

Dataset	Metrics	DistMult	NFM	KGE_NFM_nodes	KGE_NFM_nopca	KGE_NFM
Luo’s dataset	AUROC	0.814	0.923	0.931	0.913	**0.962**
	Std	0.023	0.012	0.015	0.022	0.008
	AUPR	0.426	0.743	0.762	0.648	**0.855**
	Std	0.057	0.045	0.068	0.089	0.035
Hetionet	AUROC	0.959	0.911	0.970	/	**0.972**
	Std	0.002	0.003	0.002	/	0.001
	AUPR	0.691	0.610	0.774	/	**0.780**
	Std	0.009	0.009	0.006	/	0.008
Yamanishi_08’s dataset	AUROC	0.963	0.939	0.970	/	**0.983**
	Std	0.026	0.013	0.006	/	0.004
	AUPR	0.792	0.773	0.826	/	**0.902**
	Std	0.120	0.048	0.035	/	0.019
BioKG	AUROC	0.933	0.938	0.983	/	**0.987**
	Std	0.008	0.006	0.002	/	0.001
	AUPR	0.729	0.769	0.888	/	**0.898**
	Std	0.013	0.017	0.009	/	0.007

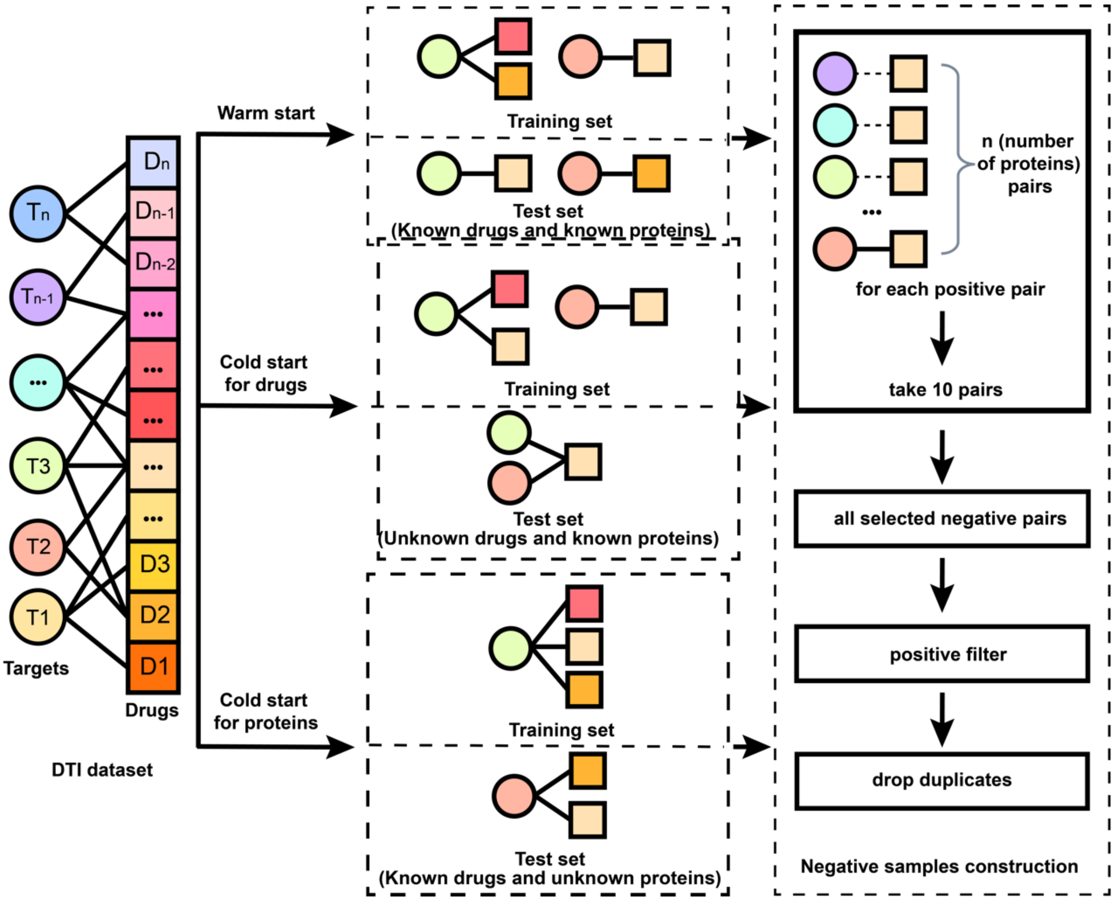

Supplementary Figure 1. Schematic diagram of dataset splitting and negative sample construction. In the scenario of the warm start, we enable the drugs and proteins in the test set are known to the training set; in the scenario of the cold start for drugs, we enable the drugs in the test set are all unknown to the training set; in the scenario of the cold start for proteins, we enable the proteins in the test set are all unknown to the training set. The construction of negative samples is same in three scenarios. Specifically, each drug in the dataset establishes the relationship pairs with all proteins that the whole benchmark dataset owns, and 10 relationship pairs are then randomly selected. The final negative samples are obtained from all the selected negative pairs after filtering positive samples and dropping duplicates.

References

1. Himmelstein, D.S., et al., *Systematic integration of biomedical knowledge prioritizes drugs for repurposing.* Elife, 2017. **6**: p. e26726.
2. Mohamed, S.K., V. Nováček, and A. Nounu, *Discovering protein drug targets using knowledge graph embeddings.* Bioinformatics, 2020. **36**(2): p. 603-610.

3. Walsh, B., S.K. Mohamed, and V. Nováček. *Biokg: A knowledge graph for relational learning on biological data*. in *Proceedings of the 29th ACM International Conference on Information & Knowledge Management*. 2020.
