# Supplementary material for: A unified drug–target interaction prediction framework based on knowledge graph and recommendation system
Source: Nat Commun. 2021 Nov 22;12:6775. doi: 10.1038/s41467-021-27137-3 (PMC8635420; doi:10.1038/s41467-021-27137-3)
Supplement: Supplementary file 3 — Reporting Summary [file 41467_2021_27137_MOESM3_ESM.pdf]

nature portfolio

Corresponding author(s): Dongsheng Cao, Shibo He, Tingjun Hou

Last updated by author(s): Nov 3, 2021

Reporting Summary

Nature Portfolio wishes to improve the reproducibility of the work that we publish. This form provides structure for consistency and transparency in reporting. For further information on Nature Portfolio policies, see our Editorial Policies and the Editorial Policy Checklist.

Statistics

For all statistical analyses, confirm that the following items are present in the figure legend, table legend, main text, or Methods section.

n/a	Confirmed	
☐	☒	The exact sample size (*n*) for each experimental group/condition, given as a discrete number and unit of measurement
☐	☒	A statement on whether measurements were taken from distinct samples or whether the same sample was measured repeatedly
☒	☐	The statistical test(s) used AND whether they are one- or two-sided<br>*Only common tests should be described solely by name; describe more complex techniques in the Methods section.*
☒	☐	A description of all covariates tested
☐	☒	A description of any assumptions or corrections, such as tests of normality and adjustment for multiple comparisons
☐	☒	A full description of the statistical parameters including central tendency (e.g. means) or other basic estimates (e.g. regression coefficient) AND variation (e.g. standard deviation) or associated estimates of uncertainty (e.g. confidence intervals)
☒	☐	For null hypothesis testing, the test statistic (e.g. *F*, *t*, *r*) with confidence intervals, effect sizes, degrees of freedom and *P* value noted<br>*Give P values as exact values whenever suitable.*
☒	☐	For Bayesian analysis, information on the choice of priors and Markov chain Monte Carlo settings
☐	☒	For hierarchical and complex designs, identification of the appropriate level for tests and full reporting of outcomes
☐	☒	Estimates of effect sizes (e.g. Cohen's *d*, Pearson's *r*), indicating how they were calculated

Our web collection on statistics for biologists contains articles on many of the points above.

Software and code

Policy information about availability of computer code

Data collection	No software was used for data collection.
Data analysis	The models (KGE_NFM, DistMult, NFM) were implemented using python (3.6) with AmpliGraph (1.3.2), DeepCTR (0.8.4) and Tensorflow (1.15.0). The model (RF), data processing and metrics calculation were implemented using python (3.6) with scikit-learn (0.24.1). The models (MPNN_CNN, DeepDTI) were implemented using python (3.6) with DeepPurpose (https://github.com/kexinhuang12345/DeepPurpose) and PyTorch (1.6.0). The source data and codes of KGE_NFM are available on the Zenodo at https://zenodo.org/record/5500305.

For manuscripts utilizing custom algorithms or software that are central to the research but not yet described in published literature, software must be made available to editors and reviewers. We strongly encourage code deposition in a community repository (e.g. GitHub). See the Nature Portfolio guidelines for submitting code & software for further information.

Data

Policy information about availability of data

All manuscripts must include a data availability statement. This statement should provide the following information, where applicable:

- Accession codes, unique identifiers, or web links for publicly available datasets
- A description of any restrictions on data availability
- For clinical datasets or third party data, please ensure that the statement adheres to our policy

The source data and data folds in the three sample scenarios used in this study are provided on the Zenodo at https://zenodo.org/record/5500305. The source data of the four benchmarks (the Luo's dataset, Hetionet, the Yamanishi_08's dataset and BioKG) is available on the https://github.com/luoyunan/DTINet, https://het.io/about/, https://drugtargets.insight-centre.org/, https://github.com/dsi-bdi/biokg, respectively.

Field-specific reporting

Please select the one below that is the best fit for your research. If you are not sure, read the appropriate sections before making your selection.

[x] Life sciences [ ] Behavioural & social sciences [ ] Ecological, evolutionary & environmental sciences

For a reference copy of the document with all sections, see nature.com/documents/nr-reporting-summary-flat.pdf

Life sciences study design

All studies must disclose on these points even when the disclosure is negative.

Sample size	No sample size calculation was performed. We evaluated our method on the four benchmarks and the KG size of the four benchmarks (the Luo's dataset, Hetionet, the Yamanishi_08's dataset and BioKG) is 1895445, 2250196, 95579 and 2043846, respectively. The consistent performance of our method through different datasets demonstrates such sample sizes are sufficient.
Data exclusions	No data were excluded from the analyses.
Replication	For each sample scenarios of each benchmark, we conducted 10-fold cross-validation, where we independently replicated the training and evaluation processes for 10 times. Since we have four benchmarks and three sample scenarios, we have replicated our model for 120 times in total. All attempts at replication were successful.
Randomization	For each benchmark, we randomly picked up about 10% data for evaluation according to the different sample scenarios. Then, the remaining data was randomly spitted into 10 folds, where one fold is for the validation and the other nine folds are for the training. In addition, the negative samples are also randomly picked as follows. For negative sample construction, each drug in the dataset establishes the relationship pairs with all proteins that the whole benchmark dataset owns, and 10 relationship pairs are then randomly selected. The final negative samples are obtained from all the selected negative pairs after filtering positive samples and dropping duplicates.
Blinding	Investigators were not blinded to group allocation during experiments and outcome analysis.

Reporting for specific materials, systems and methods

We require information from authors about some types of materials, experimental systems and methods used in many studies. Here, indicate whether each material, system or method listed is relevant to your study. If you are not sure if a list item applies to your research, read the appropriate section before selecting a response.

Materials & experimental systems

n/a	Involved in the study	
[x]	[ ]	Antibodies
[x]	[ ]	Eukaryotic cell lines
[x]	[ ]	Palaeontology and archaeology
[x]	[ ]	Animals and other organisms
[x]	[ ]	Human research participants
[x]	[ ]	Clinical data
[x]	[ ]	Dual use research of concern

Methods

n/a	Involved in the study	
[x]	[ ]	ChIP-seq
[x]	[ ]	Flow cytometry
[x]	[ ]	MRI-based neuroimaging
